# Supplementary material for: The role of collegiality in academic review, promotion, and tenure
Source: PLoS One. 2022 Apr 6;17(4):e0265506. doi: 10.1371/journal.pone.0265506 (PMC8986017; doi:10.1371/journal.pone.0265506)
Supplement: Attachment — Submitted filename: ResponseToReviewers.docx [file pone.0265506.s001.docx]

**Response to Editor and Reviewers**

Thank you for providing us with these two reviews and additional editorial suggestions. We noted with delight that Reviewer 2 provided only praise of our manuscript and had no suggestions for improvement. We thank Reviewer 2 for their positive feedback and careful read of our study.

In what follows, we provide responses to Reviewer 1’s comments and suggestions. Our responses can be found in red, while Reviewer 1’s comments are in black. We note, however, that Reviewer 1’s comments were rather vague, and in some cases difficult to interpret in light of our manuscript. For example, they ask for the Research Questions to be built into the introduction from a pertinent bibliography, which is precisely what we had done (RQs are clearly stated at the end of the introduction). Nevertheless, we have tried to address the comments to the best of our ability.

Finally, we have made the formatting changes requested. They are described below, and can be found in the manuscript with Track Changes turned on.

Sincerely,

Juan Pablo Alperin
Associate Professor, [Publishing](https://publishing.sfu.ca/)
Co-Scientific Director, [Public Knowledge Project](https://pkp.sfu.ca/)
Co-Director, [Scholarly Communications Lab](https://www.scholcommlab.ca/)
Simon Fraser University

**The role of collegiality in academic review, promotion, and tenure – Dawson et al.**

Journal Requirements:

When submitting your revision, we need you to address these additional requirements.

1. Please ensure that your manuscript meets PLOS ONE's style requirements, including those for file naming. The PLOS ONE style templates can be found at

<https://journals.plos.org/plosone/s/file?id=wjVg/PLOSOne_formatting_sample_main_body.pdf> and <https://journals.plos.org/plosone/s/file?id=ba62/PLOSOne_formatting_sample_title_authors_affiliations.pdf>

Authors’ response:

- Updated formatting of authors’ names and affiliations to adhere to PLOS ONE’s style requirements
- Re-named manuscript file to be more descriptive, though I can’t find any style requirements for file naming
- Ensured that manuscript conformed to all other body formatting guidelines

2. Thank you for stating the following in the Funding Section of your manuscript:

"Funding for this project was provided to JPA, MTN, ECM, and LAS from the Open Society Foundations (OR2018-46345). The funders had no role in study design, data collection and analysis, decision to publish, or preparation of the manuscript."

We note that you have provided funding information. However, funding information should not appear in the Funding section or other areas of your manuscript. We will only publish funding information present in the Funding Statement section of the online submission form.

**Please remove any funding-related text from the manuscript and let us know how you would like to update your Funding Statement.** Currently, your Funding Statement reads as follows:

"Funding for this project was provided to JPA, MTN, ECM, and LAS from the Open Society Foundations (OR2018-46345). The funders had no role in study design, data collection and analysis, decision to publish, or preparation of the manuscript."

**Please include your amended statements within your cover letter; we will change the online submission form on your behalf.**

Authors’ response:

- We removed this section from the manuscript.
- We have no amendments to the Funding Statement:
  - "Funding for this project was provided to JPA, MTN, ECM, and LAS from the Open Society Foundations (OR2018-46345). The funders had no role in study design, data collection and analysis, decision to publish, or preparation of the manuscript."

3. Thank you for stating the following in your Competing Interests section:

"MTN is a member of the board of directors of The Public Library of Science (PLOS). This role has in no way influenced the outcome or development of this work or the peer-review process, nor does it alter our adherence to PLOS ONE policies on sharing data and materials."

Please complete your Competing Interests on the online submission form to state any Competing Interests. If you have no competing interests, please state ""The authors have declared that no competing interests exist."", as detailed online in our guide for authors at <http://journals.plos.org/plosone/s/submit-now>

**This information should be included in your cover letter; we will change the online submission form on your behalf.**

Authors’ response:

- We removed this section from the manuscript.
- We have no amendments to the Competing Interests statement:
  - "MTN is a member of the board of directors of The Public Library of Science (PLOS). This role has in no way influenced the outcome or development of this work or the peer-review process, nor does it alter our adherence to PLOS ONE policies on sharing data and materials."

4. Please include captions for your Supporting Information files at the end of your manuscript, and update any in-text citations to match accordingly. Please see our Supporting Information guidelines for more information: <http://journals.plos.org/plosone/s/supporting-information>.

Authors’ response:

- We have removed the supporting information document (SRQR Checklist). It does not need to be published, and was only included upon submission in order to aid in the reviewing of this manuscript.

5. Please review your reference list to ensure that it is complete and correct. If you have cited papers that have been retracted, please include the rationale for doing so in the manuscript text, or remove these references and replace them with relevant current references. Any changes to the reference list should be mentioned in the rebuttal letter that accompanies your revised manuscript. If you need to cite a retracted article, indicate the article’s retracted status in the References list and also include a citation and full reference for the retraction notice.

Authors’ response:

- We have reviewed the references and made minor corrections to formatting.
- We have not cited any retracted papers.

**Response to Reviewer #1:**

1. What is the originality of this study? The authors should clarify the originality compared with other studies.

Authors’ response:

- The originality of the study is stated in the first sentence of the last paragraph of the Introduction (lines 134-137).
- We have added additional wording in that paragraph to clarify originality in direct comparison to the two most similar published papers (lines 137-142).

2. Research questions, that drive the paper, should be built in the introduction from an ongoing and pertinent bibliography (up to 2021).

Authors’ response:

- The research questions were clearly stated at the end of the Introduction (lines 149-154). We believe they are well justified, and that we cite the relevant literature specific to the topic of collegiality in RPT.
- We have now done an additional search and have found three additional relevant articles, two of which were published after we drafted the manuscript. These have been added to the introduction (Lo et al., 2022; Hammer et al., 2019; Weisbach, 2021).

3. Answer your research question in the conclusions; what did we learn compared with current, significant research (up to 2021). The authors should make explicit suggestions about how their study effects.

Authors’ response:

- Our results section was organized using the Research Questions as sub-headings, and we once again reiterate the answers throughout the discussion, where we do so in relation to the relevant published literature.
- We have also now added a recently published articles (Lo et al., 2022, and Beatson et al., 2021) to the discussion too (lines 549-554).
- We conclude the paper with suggestions on how universities might fairly address collegiality in their RPT processes.

4. The research problem is not clear, and needs to be highlighted in light of previous studies and reports.

Authors’ response:

- The research problem and questions are stated at the end of the Introduction. We have also added further clarification in the final paragraph of the Introduction (see response #1 & #2 above).
- We have added additional references up to present, including a recently published article from January 2022 (see response to #1 & #2 above).

5. Explain how this paper differs from the related ones published in the technical literature.

Authors’ response:

- The Introduction discusses the relevant literature. We added a clarification of how our study differs from the two most closely related published papers (see response to #1 above).
